# September’s Farewell

**Published:** 1911-11

**Authors:** John C. Warbrick

**Affiliations:** Chicago


For Texas Medical Journal.

### September's Farewell.

This is the month when the leaves, in their every varying tints and hues, begin to fall from the trees, blown gently hither and thither by the softly sighing autumnal breezes.

Everywhere the ground is strewn with the sere and yellow emblems of various kinds: Those of the maple, the ash, the elm, the locust, the beechnut and the apple trees are lying clustered together here and there in large numbers. It is a month of sadness and reflection, coming as it does close after the beauty and joyousness of the warm summer days, have passed away, and the breath of winter hanging like a curtain in the background is soon to enveil it with a mantle of white.

The harvest is past,—the grain has been garnered in, the grass is faded and everywhere the trees are becoming bare and forsaken. Being stripped of their foliage they stretch their naked and lonely arms abroad through the air, soon to be shaken and chilled by the cold winter blasts. A few stray leaves still cling to some of the branches on a few of the trees until the last, as if to say: "We regret to depart, but to fall and fade and die as decreed by nature we must."

In all her garb of beauty, September now is soon to pass away,—

only to be followed by the bleak and cheerless October days. The glorious sunshine, the busy insects, the chirp of the crickets of the golden autumnal days, will soon be no more. The melody of the birds is no longer heard amongst the leafy trees as they flitted from limb to limb, pealing forth their merry notes.

Everywhere the air is still save for the soft rustling of the leaves upon the ground, which crackle 'neath the tread of one's feet. Hardly a limb appears to move, and nature, in her lovely tinted raiment,—almost seems to sleep in calm repose. Now and then the quick shirring of a red squirrel is heard, as it frisks around the tree trunks or climbs quickly up to a branch and breaks upon the solemn silence;—then again the cawing of the crows floats upon the air, as at a great height in the distance they can be seen sailing over the tree-tops together, crying, "caw, caw, caw," making the scene more picturesque and the occasion more beautiful to behold. —John C. Warbrick, M. D., Chicago.

